# Evaluation of disease severity and prediction of severe cases in children hospitalized with influenza A (H1N1) infection during the post-COVID-19 era: a multicenter retrospective study

**DOI:** 10.1186/s12887-024-04645-x

**Published:** 2024-04-02

**Authors:** Hai-Feng Liu, Xiao-Zhong Hu, Rong-Wei Huang, Zheng-Hong Guo, Jin-Rong Gao, Mei Xiang, Rui Lu, Deng Ban, Cong-Yun Liu, Ya-Yu Wang, Wang Li, Yin Li, Yun-Jie Guo, Quan Lu, Hong-Min Fu

**Affiliations:** 1 Department of Pulmonary and Critical Care Medicine, Yunnan Key Laboratory of Children’s Major Disease Research, Yunnan Medical Center for Pediatric Diseases, Kunming Children’s Hospital, Kunming Medical University, No. 28, Shulin Street, Xishan District, Kunming, 650034 China; 2 Department of Pediatrics, The People’s Hospital of Lincang, Lincang, 677000 China; 3 Department of Pediatrics, Zhaotong Hospital Affiliated to Kunming Medical University, Zhaotong, 657000 China; 4 Department of Pediatrics, The First People’s Hospital of Honghe, Honghe, 651400 China; 5 Department of Pediatrics, The People’s Hospital of Wenshan, Wenshan, 663000 China; 6 Department of Pediatrics, The People’s Hospital of Baoshan, Baoshan, 678000 China; 7 Department of Pediatrics, The Third Affiliated Hospital of Dali University, Dali, 671000 China; 8 Department of Pediatrics, The Fifth People’s Hospital of Kunming, Kunming, 650200 China; 9 Department of Pulmonary Medicine, Shanghai Children’s Hospital, Shanghai Jiao Tong University, No. 1400 West Beijing Road, Jinan District, Shanghai, 200040 China

**Keywords:** Influenza A (H1N1) infection, Children, Post-COVID-19 era, Prediction model, Severe cases

## Abstract

**Background:**

The rebound of influenza A (H1N1) infection in post-COVID-19 era recently attracted enormous attention due the rapidly increased number of pediatric hospitalizations and the changed characteristics compared to classical H1N1 infection in pre-COVID-19 era. This study aimed to evaluate the clinical characteristics and severity of children hospitalized with H1N1 infection during post-COVID-19 period, and to construct a novel prediction model for severe H1N1 infection.

**Methods:**

A total of 757 pediatric H1N1 inpatients from nine tertiary public hospitals in Yunnan and Shanghai, China, were retrospectively included, of which 431 patients diagnosed between February 2023 and July 2023 were divided into post-COVID-19 group, while the remaining 326 patients diagnosed between November 2018 and April 2019 were divided into pre-COVID-19 group. A 1:1 propensity-score matching (PSM) was adopted to balance demographic differences between pre- and post-COVID-19 groups, and then compared the severity across these two groups based on clinical and laboratory indicators. Additionally, a subgroup analysis in the original post-COVID-19 group (without PSM) was performed to investigate the independent risk factors for severe H1N1 infection in post-COIVD-19 era. Specifically, Least Absolute Shrinkage and Selection Operator (LASSO) regression was applied to select candidate predictors, and logistic regression was used to further identify independent risk factors, thus establishing a prediction model. Receiver operating characteristic (ROC) curve and calibration curve were utilized to assess discriminative capability and accuracy of the model, while decision curve analysis (DCA) was used to determine the clinical usefulness of the model.

**Results:**

After PSM, the post-COVID-19 group showed longer fever duration, higher fever peak, more frequent cough and seizures, as well as higher levels of C-reactive protein (CRP), interleukin 6 (IL-6), IL-10, creatine kinase-MB (CK-MB) and fibrinogen, higher mechanical ventilation rate, longer length of hospital stay (LOS), as well as higher proportion of severe H1N1 infection (all *P* < 0.05), compared to the pre-COVID-19 group. Moreover, age, BMI, fever duration, leucocyte count, lymphocyte proportion, proportion of CD3^+^ T cells, tumor necrosis factor α (TNF-α), and IL-10 were confirmed to be independently associated with severe H1N1 infection in post-COVID-19 era. A prediction model integrating these above eight variables was established, and this model had good discrimination, accuracy, and clinical practicability.

**Conclusions:**

Pediatric H1N1 infection during post-COVID-19 era showed a higher overall disease severity than the classical H1N1 infection in pre-COVID-19 period. Meanwhile, cough and seizures were more prominent in children with H1N1 infection during post-COVID-19 era. Clinicians should be aware of these changes in such patients in clinical work. Furthermore, a simple and practical prediction model was constructed and internally validated here, which showed a good performance for predicting severe H1N1 infection in post-COVID-19 era.

**Graphical Abstract:**

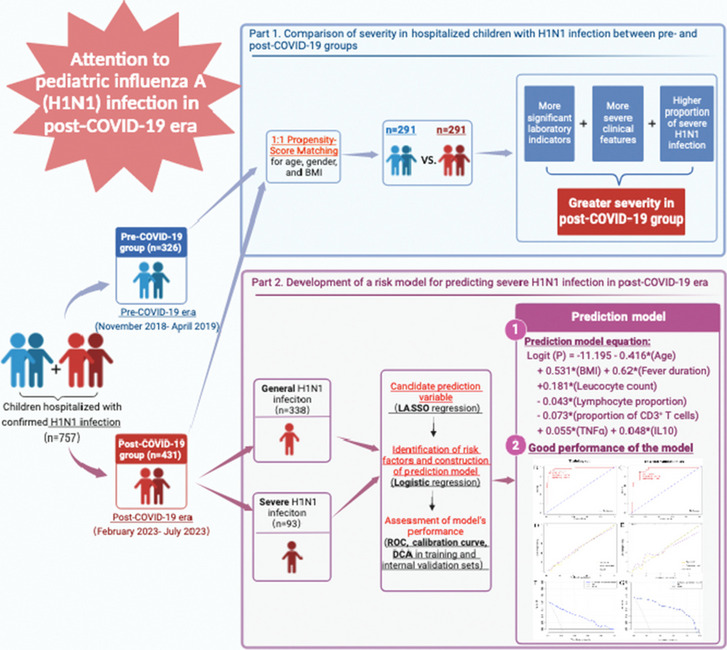

## Introduction

Influenza has been one of the major concerns encountered in the public health domain. Populations of all ages are susceptible to infection with influenza viruses, especially children due to their underdeveloped immune defense mechanisms [1]. According to statistics, approximately 109.5 million children under 5 years of age were infected with influenza viruses in 2018. Of these, 870,000 children were hospitalized with influenza-related acute lower respiratory infection (ALRI) and up to 34,800 deaths, accounting for 4% of all ALRI-related deaths in this age group [2]. However, it is noteworthy that during the COVID-19 pandemic, both Northern and Southern Hemispheres showed little records of influenza activity. This phenomenon was primarily attributed to the implementation of non-pharmaceutical interventions (NPIs), including use of face masks, social distancing, etc., which not only limited the transmission of SARS-CoV-2 but also influenza viruses [3–5]. Despite this positive collateral effect on preventing additional overload of the healthcare system in the short term, the lack of immune stimulation due to the declined circulation of pathogens induced an “immunity debt” which may result in negative consequences when the COVID-19 pandemic is controlled and NPIs are lifted [6, 7]. In fact, in the post- COVID-19 era with the lifting of strict NPIs, many countries and regions have reported significant resurgence of influenza circulation [8, 9]. Influenza rebound following three seasons of inactivity was also observed in China after gradually easing NPIs. According to the data from Chinese Center for Disease Control and Prevention (CDC) (https://www.chinacdc.cn/), a rapid increase in the number of pediatric patients hospitalized with influenza A (H1N1) infection was observed in February 2023, and the positive rate of H1N1 infection peaked (53.2%) in 10th week of 2023 (February 6, 2023- February 12, 2023). Newly diagnosed cases were still being reported every day as of July 2023 in various regions of China, including Yunnan.

On the other hand, the history of SARS-CoV-2 infection may exert a detrimental clinical influence on H1N1 infection in the post-COVID-19 era. During the latest Omicron wave between December 2022 and February 2023 in China, over 82% of the Chinese population contracted SARS-CoV-2 Omicron infection between December 2022 and February 2023 [10]. One or more persistent COVID-19-related symptoms with hypothesised pathophysiologic mechanisms involving residual virus in a variety of tissues, immune dysregulation, and autoimmunity resulting from cross reactivity of SARS-CoV-2-specific antibodies with host proteins, etc. have been reported in a significant proportion of individuals who recovered from COVID-19 for months or even longer [11–14]. Therefore, the prior infection of SARS-CoV-2 is a non-negligible factor that may further exacerbate the clinical severity and manifestations of children with H1N1 infection in the post-COVID-19 era.

Until now, most studies about influenza infection in post-COIVD-19 era focused on the epidemiological investigations but limited studies concentrated on the changes in clinical severity and presentation of influenza infection in children. In this study, we retrospectively collected demographic and clinical data from children hospitalized with H1N1 during November 2018- April 2019 (influenza season in Northern Hemisphere before COVID-19 pandemic) and February 2023- July 2023 (post-COVID-19 era) in nine hospitals in Yunnan and Shanghai, investigating the changes in clinical severity and characteristics of pediatric H1N1 infection from pre- to post-COVID-19 era and constructing a predictive model for severe H1N1 cases in the post-COVID-19 era.

## Methods

### Participants

This multicenter retrospective study was performed at nine hospitals in Yunnan and Shanghai, all of which are large, public tertiary hospitals. Children who were hospitalized with H1N1 infection in any of the participating hospitals during November 2018- April 2019 and February 2023- July 2023 were retrospectively included in our study. The inclusion criteria were as follows: (1) children who met the diagnostic criteria of influenza A (H1N1) infection [15, 16]; (2) children aged ≤ 14 years at initial diagnosis; and (3) the interval from symptom onset to admission was within 48 h. All subjects eventually included were divided into two groups, including pre-COVID-19 group (November 2018- April 2019) and post-COVID-19 group (February 2023- July 2023), based on the date of admission.

According to the World Health Organization (WHO) guidelines for influenza A (H1N1) virus [17], severe H1N1 infection were defined as patients presenting with one or more of the following manifestations: (1) dyspnea, tachypnea, or hypoxia; (2) radiological signs of lower respiratory tract disease (e.g. pneumonia); (3) severe central nervous system (CNS) involvement (e.g., encephalopathy, encephalitis); (4) severe dehydration; (5) multiorgan failure; (6) septic shock; (7) exacerbation of underlying chronic disease, including asthma, chronic obstructive pulmonary disease, chronic hepatic or renal insufficiency, diabetes mellitus, or other cardiovascular conditions; and (8) any other influenza-related condition or clinical manifestation requiring hospital admission. The remaining cases were defined as the general H1N1 infection.

This study was performed under the Declaration of Helsinki and was approved by the Ethics Committees of Kunming Children’s Hospital Affiliated to Kunming Medical University (the lead institution of this study) (approval number: 2023-04-129-K01), who also waived the informed consent due to the retrospective design of the study.

### Data extraction

A retrospective review of subjects’ medical records at admission from the participating hospitals was conducted to gather the demographic information [age, gender, body mass index (BMI)], clinical features (fever, fever duration, fever peak, cough, rhinorrhea, wheezing, sore throat, headache, myalgia, seizures, drowsiness, diarrhea, vomiting, abdominal pain), laboratory characteristics [leucocyte count, neutrophil proportion, lymphocyte proportion, proportions of CD3^+^ T cells, CD3^+^8^+^ T cells, CD3^+^4^+^ T cells, NK cells and B cells, C-reactive protein (CRP), interleukin 6 (IL-6), IL-10, tumor necrosis factor α (TNF-α), creatine kinase-MB (CK-MB), high-sensitivity troponin T (hs-TnT), alanine transaminase (ALT), aspartate transaminase (AST), fibrinogen], co-infection with SARS-CoV-2, mechanical ventilation rate, length of hospital stay (LOS), as well as rate of severe H1N1 infection. The vaccination information including data on H1N1 vaccination and COVID-19 vaccination status was also collected and patients who received at least one dose were considered as vaccinated. All data were reviewed and checked by a trained team of clinicians.

### Statistical analysis

The whole study was divided into two parts: (1) comparison of clinical severity across the pre- and post-COVID-19 groups and (2) construction of prediction model for severe pediatric H1N1 infection.

In the part 1, to minimize the influence of potential demographic confounders, propensity-score matching (PSM) using 1:1 nearest neighbor matching with a caliper of 0.02 was adopted to balance age, gender, and BMI between these two groups, and then clinical severity was compared between groups based on a series of clinical and laboratory characteristics.

In the part 2, a subgroup analysis in the original post-COVID-19 group (without PSM) was performed to construct a model for predicting the risk of severe pediatric H1N1 infection during the post-COVID-19 era. Specifically, during this process, to avoid omitting important predictive variable, all variables (except for mechanical ventilation rate and LOS) were included into Least Absolute Shrinkage and Selection Operator (LASSO) regression, which was utilized to minimize potential multicollinearity and overfitting, thus selecting most useful candidate predictors based on the value of lambada. To ensure the accuracy and conciseness of the model at the same time, a lambda within one standard error of the minimum criteria (lambda.1se) was chosen as the optimal lambda by a 10-fold cross-validation using the built-in function *cv.glmnet* from R package *glmnet*. Logistic regression analysis was used to further identify independent risk factors, establishing the prediction model. Patients in the post-COVID-19 group were randomly divided into training and internal validation sets at a ratio of 7:3 using the R package *caret*, then the receiver operating characteristic (ROC) curve (R package *pROC*), calibration curve (R package *rms*), and decision curve analysis (DCA) (R package *rmda*) were used in both training and internal validation sets for assessing the discriminatory ability, accuracy, and clinical benefit of this model, respectively.

In addition, general descriptive statistics were calculated as follows. Distribution of variables was checked using the Shapiro-Wilk test. Categorical variables were presented as percentages (%) and compared using Pearson’s chi-square or Fisher’s exact test, while continuous variables with skewed distribution were expressed as medians (interquartile range, IQR) and compared using Mann-Whitney U test. All statistical analyses were conducted using R software version 3.5.1 (R Foundation, Vienna, Austria) with a two-side significance level of *P* value < 0.05.

## Results

### Comparison of patients’ characteristics and severity between the pre- and post-COVID-19 groups after PSM

**Fig. 1:**
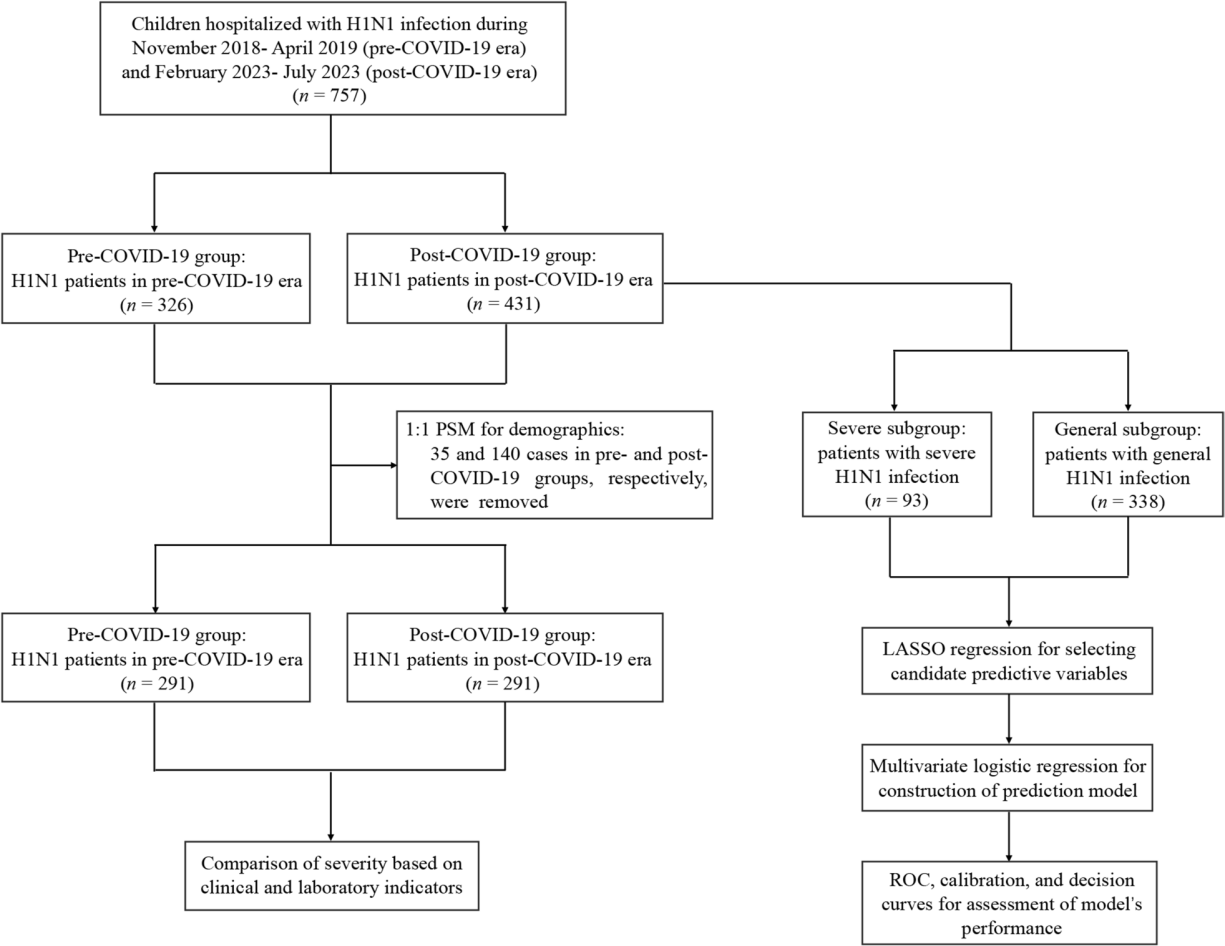
Study design and patient flow-chart within the study

During the entire study period, a total of 757 children with H1N1 infection were included. Among these, 431 and 326 patients were classified into the post- and pre-COVID-19 groups, respectively (Fig. 1). As shown in Table 1, prior to PSM, the post-COVID-19 group showed an older median age at infection (5.4 vs. 4.6 years, *P* = 0.001), a slighter male predominance (58.0% vs. 66.0%, *P* = 0.026), as well as a higher BMI (18.0 vs. 17.1 kg/m^2^, *P* < 0.001), compared to the pre-COVID-19 group. After PSM, 291 subjects were retained in each group with no significant differences in distribution of age, gender, and BMI (all *P* > 0.05).

**Table 1:** Characteristics of participants in pre- and post-COVID-19 groups before and after PSM

Characteristics	Before PSM			After PSM		
	Post-COVID-19 (*n* = 431)	Pre-COVID-19 (*n* = 326)	* P* value	Post-COVID-19 (*n* = 291)	Pre-COVID-19 (*n* = 291)	* P* value
Age (y), median (IQR)	5.4 (3.6, 6.5)	4.6 (3.3, 5.9)	0.001	5.1 (3.5, 6.3)	4.7 (3.3, 6.1)	0.236
Male, n (%)	250 (58.0)	215 (66.0)	0.026	190 (65.3%)	183 (62.9%)	0.545
BMI (kg/m^2^), median (IQR)	18.0 (16.5, 19.5)	17.1 (15.5, 18.9)	< 0.001	17.6 (16.3, 19.1)	17.3 (15.9, 19.1)	0.470
Vaccination status, n (%)						
H1N1 vaccination	16 (3.7)	9 (2.8)	0.468	10 (3.4)	6 (2.1)	0.311
COVID-19 vaccination	400 (92.8)	N/A	N/A	N/A	N/A	N/A
One-dose vaccination	89 (20.6)	N/A	N/A	N/A	N/A	N/A
Two-dose vaccination	201 (46.6)	N/A	N/A	N/A	N/A	N/A
Three-dose vaccination	110 (25.5)	N/A	N/A	N/A	N/A	N/A
Symptoms, n (%)						
Fever	420 (97.4)	319 (97.9)	0.717	282 (96.9)	285 (97.9)	0.433
Duration of fever (d), median (IQR)	4.0 (3.0, 6.0)	2.5 (2.0, 4.0)	< 0.001	4.0 (3.0, 6.0)	3.0 (2.0, 4.0)	< 0.001
Fever peak (°C), median (IQR)	39.0 (38.7, 39.4)	38.8 (38.6, 39.3)	< 0.001	39.0 (38.7, 39.4)	38.8 (38.6, 39.3)	< 0.001
Cough	395 (91.6)	273 (83.7)	0.001	264 (90.7)	240 (82.5)	0.003
Rhinorrhea	157 (36.4)	114 (35.0)	0.679	106 (36.4)	99 (34.0)	0.544
Wheezing	131 (30.4)	75 (23.0)	0.024	80 (27.5)	67 (23.0)	0.215
Sore throat	83 (19.3)	61 (18.7)	0.850	55 (18.9)	59 (20.3)	0.676
Headache	49 (11.4)	33 (10.1)	0.585	28 (9.6)	28 (9.6)	1.000
Myalgia	105 (24.4)	86 (26.4)	0.527	75 (25.8)	74 (25.4)	0.924
Seizures	78 (18.1)	23 (7.1)	< 0.001	53 (18.2)	22 (7.6)	< 0.001
Drowsiness	150 (34.8)	104 (31.9)	0.403	96 (33.0)	98 (33.7)	0.860
Diarrhea	49 (11.4)	34 (10.4)	0.682	29 (10.0)	32 (11.0)	0.685
Vomiting	62 (14.4)	50 (15.3)	0.715	37 (12.7)	47 (16.2)	0.238
Abdominal pain	32 (7.4)	21 (6.4)	0.600	22 (7.6)	18 (6.2)	0.512
Laboratory findings, median (IQR)						
Leukocyte count (×10^9^/L)	6.3 (4.3, 9.0)	6.0 (4.3, 8.5)	0.341	6.1 (4.2, 8.7)	6.0 (4.3, 8.7)	0.707
Neutrophil proportion (%)	47.0 (31.0, 60.7)	47.4 (31.8, 61.0)	0.953	46.5 (30.7, 60.2)	47.4 (32.4, 61.2)	0.623
Lymphocyte proportion (%)	41.5 (28.0, 56.4)	40.6 (28.2, 55.1)	0.921	41.8 (29.6, 58.3)	40.5 (27.6, 55.1)	0.474
Proportion of CD3^+^T cells (%)	69.7 (64.2, 73.2)	68.3 (64.8, 72.2)	0.198	69.8 (65.6, 73.2)	68.1 (64.1, 71.7)	0.126
Proportion of CD3^+^8^+^T cells (%)	25.4 (21.3, 31.1)	26.2 (21.5, 31.0)	0.454	25.3 (21.2, 30.5)	26.4 (22.3, 31.0)	0.108
Proportion of CD3^+^4^+^T cells (%)	35.8 (28.3, 42.3)	36.7 (30.9, 42.4)	0.189	36.3 (30.0, 42.4)	36.7 (31.1, 42.4)	0.544
Proportion of NK cells (%)	12.7 (9.1, 16.6)	12.1 (8.5, 15.6)	0.071	12.7 (9.0, 16.4)	12.2 (8.5, 15.6)	0.208
Proportion of B cells (%)	18.9 (15.2, 22.5)	18.4 (13.9, 22.8)	0.417	18.7 (15.6, 22.5)	18.5 (14.0, 23.0)	0.665
CRP (mg/L)	15.4 (12.3, 23.1)	11.7 (9.1, 14.6)	< 0.001	14.9 (12.5, 18.2)	11.7 (8.8, 14.5)	< 0.001
IL-6 (pg/mL)	20.8 (16.9, 31.6)	11.1 (5.6, 20.4)	< 0.001	20.5 (16.9, 30.1)	11.0 (5.6, 21.0)	< 0.001
IL-10 (pg/mL)	16.6 (12.7, 22.3)	15.0 (12.0, 20.5)	0.020	16.5 (12.8, 22.2)	14.9 (11.9, 20.6)	0.030
TNF-α (pg/mL)	17.9 (14.0, 23.0)	16.8 (12.8, 22.5)	0.027	18.0 (13.8, 22.8)	17.1 (13.3, 22.7)	0.161
CK-MB (U/L)	44.9 (40.1, 53.1)	36.0 (30.4, 42.8)	< 0.001	44.9 (40.7, 52.0)	36.0 (30.2, 42.8)	< 0.001
hs-TnT (pg/mL)	4.6 (3.0, 6.9)	4.3 (2.9, 7.0)	0.401	4.7 (3.0, 6.9)	4.3 (2.9, 7.2)	0.433
ALT (U/L)	16.0 (12.0, 23.0)	17.0 (12.0, 26.0)	0.098	16.0 (12.0, 23.0)	17.0 (12.0, 26.0)	0.125
AST (U/L)	32.0 (24.0, 41.0)	33.0 (24.0, 43.0)	0.290	32.0 (23.0, 42.0)	33.0 (24.0, 43.0)	0.345
Fibrinogen (g/L)	3.9 (3.5, 4.6)	3.7 (3.2, 4.6)	0.005	3.9 (3.5, 4.7)	3.7 (3.2, 4.7)	0.046
Co-infection with SARS-CoV-2, n (%)	55 (12.8)	N/A	N/A	N/A	N/A	N/A
Mechanical ventilation, n (%)	20 (4.6)	4 (1.2)	0.008	12 (4.1)	4 (1.4)	0.043
LOS (d), median (IQR)	8.0 (7.0, 9.0)	6.0 (5.0, 7.0)	< 0.001	8.0 (7.0, 9.0)	6.0 (5.0, 7.0)	< 0.001
Severe H1N1 infection, n (%)	93 (21.6)	42 (12.9)	0.002	60 (20.6)	41 (14.1)	0.038

*ALT *alanine aminotransferase, *AST *aspartate aminotransferase, *BMI *body mass index, *CK-MB *creatine kinase-MB, *CRP *C-reactive protein, *hs-TnT *high-sensitivity troponin-T, *IL-6 *interleukin 6, *IL-10 *interleukin 10, *LOS *Length of hospital stay, *N/A *not applicable, *PSM * propensity-score matching, *TNF-α *tumor necrosis factor α

According to the analysis results of the matched data, notable differences in several clinical characteristics and laboratory indicators were identified between these two groups. Specifically, compared to the pre-COVID-19 group, the post-COVID-19 group revealed markedly longer duration of fever (4.0 vs. 3.0 days, *P* < 0.001), higher fever peak (39.0 vs. 38.8 °C, *P* < 0.001), more frequent cough (90.7% vs. 82.5%, *P* = 0.003) and seizures (18.2% vs. 7.6%, *P* < 0.001), as well as higher levels of CRP (14.9 vs. 11.7 mg/L, *P* < 0.001), IL-6 (20.5 vs. 11.0 pg/mL, *P* < 0.001), IL-10 (16.5 vs. 14.9 pg/mL, *P* = 0.03), CK-MB (44.9 vs. 36.0 U/L, *P* < 0.001) and fibrinogen (3.9 vs. 3.7 g/L, *P* = 0.046). Furthermore, more patients requiring mechanical ventilation (4.1% vs. 1.4%, *P* = 0.043), longer LOS (8.0 vs. 6.0 days, *P* < 0.001), as well as a higher proportion of severe H1N1 infection (20.6% vs. 14.1%, *P* = 0.038) were found in the post-COVID-19 group than those in the pre-COVID-19 group. These findings suggested a greater severity of H1N1 infection in the post-COVID-19 group than the pre-COVID-19 group.

### Characteristics of patients in the post-COVID-19 group without PSM

There was a total of 431 patients in the post-COVID-19 group without PSM, of which 93 (21.6%) eventually developed severe H1N1 infection (severe H1N1 subgroup) and 338 (78.4%) were considered to be general type (general H1N1 subgroup). As described in Table 2, in comparison with general H1N1 cases, patients with severe H1N1 infection reported a younger age (4.0 vs. 5.5 years, *P* < 0.001), more clear male predominance (71.0% vs. 54.4%, *P* = 0.004), as well as a greater BMI (19.4 vs. 17.5 kg/m^2^, *P* < 0.001). Despite no marked between-group differences in total number of patients receiving the COVID-19 vaccine or H1N1 vaccine, the severe H1N1 subgroup indicated a lower proportion of three-dose COVID-19 vaccination than the general subgroup (17.2% vs. 27.8%, *P* = 0.038). Significant differences in clinical and laboratory characteristics were observed across these two subgroups, including longer fever duration (6.0 vs. 4.0 days, *P* < 0.001), higher fever peak (39.1 vs. 39.0 °C, *P* = 0.022), more frequent wheezing (55.9% vs. 23.4%, *P* < 0.001), seizures (44.1% vs. 10.9%, *P* < 0.001) and drowsiness (69.9% vs. 25.1%, *P* < 0.001), increased leucocyte count (9.5 vs. 5.7 × 10^9^/L, *P* < 0.001) and neutrophil proportion (56.1% vs. 44.5%, *P* < 0.001), higher levels of CRP (23.5 vs. 14.6 mg/L, *P* < 0.001), IL-6 (28.7 vs. 19.3 pg/mL, *P* < 0.001), IL-10 (23.7 vs. 14.9 pg/mL, *P* < 0.001), TNF-α (13.2 vs. 7.3 pg/mL, *P* < 0.001), CK-MB (53.2 vs. 43.9 U/L, *P* < 0.001), hs-TnT (7.8 vs. 4.1 pg/mL, *P* < 0.001) and fibrinogen (4.1 vs. 3.8 g/L, *P* = 0.002), elevated rates of co-infection with SRAS-CoV-2 (19.4% vs. 10.9%, *P* = 0.031) and mechanical ventilation (21.5% vs. 0, *P* < 0.001), as well as longer LOS (9.0 vs. 7.0 days, *P* < 0.001) in the severe subgroup than those in the general subgroup. On the other hand, the severe subgroup indicated lower proportions of lymphocyte (28.8% vs. 44.0%, *P* < 0.001), CD3^+^ T cells (58.5% vs. 70.6%, *P* < 0.001), CD3^+^8^+^ T cells (22.3% vs. 26.9%, *P* < 0.001) and CD3^+^4^+^ T cells (28.7% vs. 37.7%, *P* < 0.001), compared to the general subgroup.

**Table 2:** Characteristics of participants in the severe and general H1N1 subgroups of the post-COVID-19 group

Characteristics	Severe H1N1 subgroup (*n* = 93)	General H1N1 subgroup (*n* = 338)	* P* value
Age (y), median (IQR)	4.0 (2.3, 5.9)	5.5 (4.1, 6.6)	< 0.001
Male, n (%)	66 (71.0)	184 (54.4)	0.004
BMI (kg/m2), median (IQR)	19.4 (18.1, 21.1)	17.5 (16.3, 19.1)	< 0.001
Vaccination status, n (%)			
H1N1 vaccination	4 (4.3)	12 (3.6)	0.735
COVID-19 vaccination	85 (91.4)	315 (93.2)	0.552
One-dose vaccination	21 (22.6)	68 (20.1)	0.603
Two-dose vaccination	48 (51.6)	153 (45.3)	0.277
Three-dose vaccination	16 (17.2)	94 (27.8)	0.038
Symptoms, n (%)			
Fever	93 (100.0)	327 (96.7)	0.078
Duration of fever (d), median (IQR)	6.0 (5.0, 8.0)	4.0 (3.0, 5.0)	< 0.001
Fever peak (°C), median (IQR)	39.1 (38.8, 39.6)	39.0 (38.7, 39.4)	0.022
Cough	88 (94.6)	307 (90.8)	0.241
Rhinorrhea	36 (38.7)	121 (35.8)	0.605
Wheezing	52 (55.9)	79 (23.4)	< 0.001
Sore throat	19 (20.4)	64 (18.9)	0.746
Headache	12 (12.9)	37 (10.9)	0.599
Myalgia	17 (18.3)	88 (26.0)	0.123
Seizures	41 (44.1)	37 (10.9)	< 0.001
Drowsiness	65 (69.9)	85 (25.1)	< 0.001
Diarrhea	15 (16.1)	34 (10.1)	0.102
Vomiting	18 (19.4)	44 (13.0)	0.123
Abdominal pain	10 (10.8)	22 (6.5)	0.167
Laboratory findings, median (IQR)			
Leukocyte count (×10^9^/L)	9.5 (6.9, 12.4)	5.7 (4.1, 7.4)	< 0.001
Neutrophil proportion (%)	56.1 (45.9, 68.2)	44.5 (29.3, 57.4)	< 0.001
Lymphocyte proportion (%)	28.8 (15.3, 42.2)	44.0 (30.9, 59.7)	< 0.001
Proportion of CD3^+^T cells (%)	58.5 (47.4, 66.8)	70.6 (67.4, 74.2)	< 0.001
Proportion of CD3^+^8^+^T cells (%)	22.3 (16.7, 26.6)	26.9 (22.8, 32.1)	< 0.001
Proportion of CD3^+^4^+^T cells (%)	28.7 (23.3, 34.6)	37.7 (31.6, 43.5)	< 0.001
Proportion of NK cells (%)	13.2 (9.3, 19.7)	12.6 (9.1, 16.2)	0.199
Proportion of B cells (%)	19.3 (14.0, 29.5)	18.8 (15.8, 21.6)	0.086
CRP (mg/L)	23.5 (23.1, 25.1)	14.6 (10.1, 17.0)	< 0.001
IL-6 (pg/mL)	28.7 (19.5, 48.9)	19.3 (16.6, 28.4)	< 0.001
IL-10 (pg/mL)	23.7 (17.6, 29.0)	14.9 (12.0, 19.6)	< 0.001
TNF-α (pg/mL)	13.2 (9.5, 18.8)	7.3 (4.5, 12.7)	< 0.001
CK-MB (U/L)	53.2 (43.9, 74.4)	43.9 (39.5, 50.0)	< 0.001
hs-TnT (pg/mL)	7.8 (4.2, 13.2)	4.1 (2.6, 5.8)	< 0.001
ALT (U/L)	17.0 (13.0, 25.5)	15.0 (12.0, 21.3)	0.065
AST (U/L)	33.0 (26.0, 41.5)	32.0 (23.0, 41.0)	0.478
Fibrinogen (g/L)	4.1 (3.7, 4.9)	3.8 (3.4, 4.6)	0.002
Co-infection with SARS-CoV-2, n (%)	18 (19.4)	37 (10.9)	0.031
Mechanical ventilation, n (%)	20 (21.5)	0 (0.0)	< 0.001
LOS (d), median (IQR)	9.0 (8.0, 11.0)	7.0 (6.0, 8.0)	< 0.001

*ALT *alanine aminotransferase, *AST *aspartate aminotransferase, *BMI *body mass index, *CK-MB *creatine kinase-MB, *CRP *C-reactive protein, *hs-TnT *high-sensitivity troponin-T, *IL-6 *interleukin 6, *IL-10 *interleukin 10, *LOS *length of hospital stay, *TNF-α *tumor necrosis factor α

### Identification of independent risk factors and construction of prediction model for severe H1N1 infection in the post-COVID-19 era

**Fig. 2:**
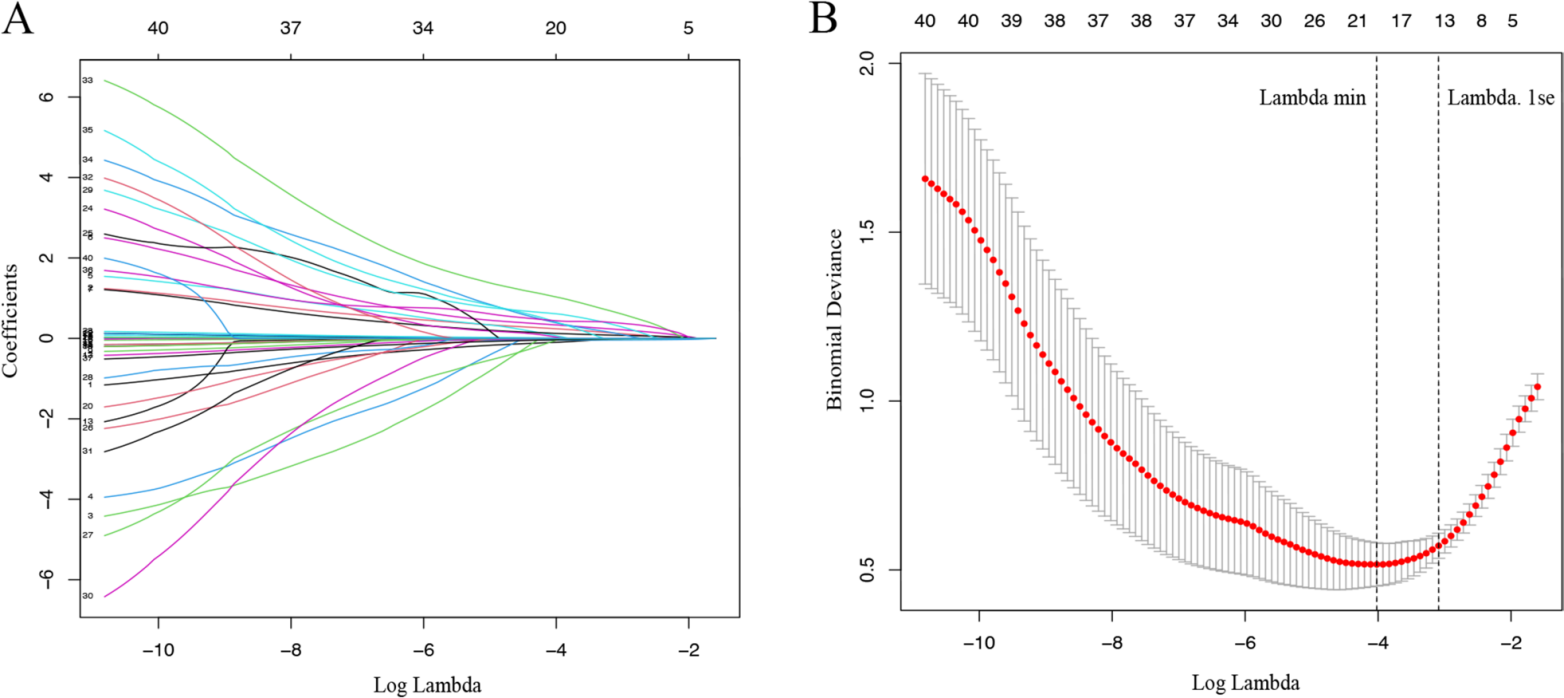
Selection of candidate predictive variables using LASSO regression. **A** LASSO coefficient profiles of these all potential predictive variables. The coefficient profile plot was generated against the log lambda sequence. **B** Tuning parameter (lambda) selection in the LASSO model using 10-fold cross-validation via one standard error of the minimum criteria (lambda.1se). Based on lambda.1se, 14 variables with non-zero coefficients were selected. LASSO, Least Absolute Shrinkage and Selection Operator

**Fig. 3:**
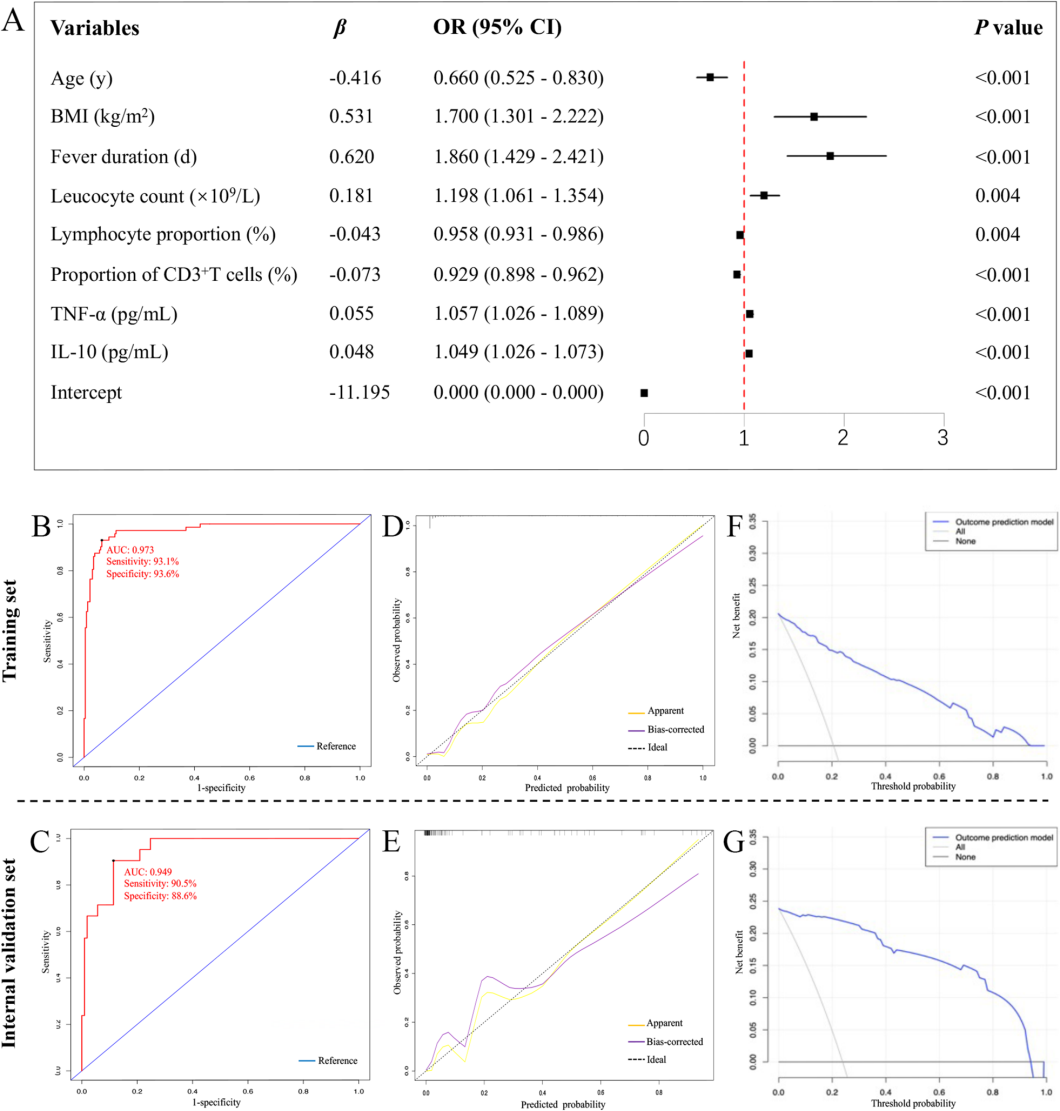
Construction and performance evaluation of the prediction model for severe pediatric H1N1 infection in post-COVID-19 era. **A** The result of logistic regression analysis. Age, BMI, fever duration, leucocyte count, lymphocyte proportion, proportion of CD3^+^ T cells, TNF-α, and IL-10 were independently associated with severe H1N1 infection in the post-COVID-19 group. **B** and **C** ROC curve analysis showed that the AUC for the training set was 0.973, with a sensitivity of 93.1% and a specificity of 93.6%, and the AUC was 0.949 for the validation set, with a sensitivity of 90.5% and a specificity of 88.6%. **D** and **E** Calibration curve analysis indicated favorable agreement between the predicted probability and the observed probability in both training and internal validation sets. **F** and **G** DCA identified good clinical utility of this prediction model in both training and internal validation sets. AUC, area under the curve; BMI, body mass index; DCA, decision curve analysis; IL-10, interleukin 10; ROC, receiver operating characteristic; TNF-α, tumor necrosis factor α

Fourteen candidate predictor variables, including age, BMI, fever duration, leucocyte count, lymphocyte proportion, proportion of CD3^+^ T cells, CRP, TNF-α, IL-10, wheezing, seizures, diarrhea, vomiting, and drowsiness, were selected by LASSO regression (Fig. 2a and b) and were further included into the logistic regression analysis. Eventually, age (OR: 0.660; 95% CI: 0.525–0.830), BMI (OR: 1.700; 95% CI:1.301–2.222), fever duration (OR: 1.860; 95% CI: 1.429–2.421), leucocyte count (OR: 1.198; 95% CI: 1.061–1.354), lymphocyte proportion (OR: 0.958; 95% CI: 0.931–0.986), proportion of CD3^+^ T cells (OR: 0.929; 95% CI: 0.898–0.962), TNF-α (OR: 1.057; 95% CI: 1.026–1.089), and IL-10 (OR: 1.049; 95% CI: 1.026–1.073) were confirmed to be the independent risk factors for severe H1N1 infection in the post-COVID-19 group (Fig. 3a). Based on the regression coefficient (*β*) values of intercept and these eight independent factors, the predicted probability equation was derived as follows:

$$\mathrm{Logit}\;(P)\:=\:-\:11.195\:-\:0.416\;\times\;\mathrm{Age}\;(\mathrm y)\:+\:0.531\;\times\;\mathrm{BMI}\;(\mathrm{kg}/\mathrm m2)\:+\:0.62\;\times\;\mathrm{Fever}\;\mathrm{duration}\;(\mathrm d)\:+\:0.181\;\times\;\mathrm{Leucocyte}\;\mathrm{count}\;(\times109/\mathrm L)\:-\:0.043\;\times\;\mathrm{Lymphocyte}\;\mathrm{proportion}\;(\%)\:-\:0.073\;\times\;\mathrm{Proportion}\;\mathrm{of}\;\mathrm{CD}3+\;\mathrm T\;\mathrm{cells}\;(\%)\:+\:0.055\;\times\;\mathrm{TNF}-\mathrm\alpha\;(\mathrm{pg}/\mathrm{mL})\:+\:0.048\;\times\;\mathrm{IL}-10\;(\mathrm{pg}/\mathrm{mL})$$

### Evaluation of model performance

ROC curves and calibration curves were plotted to evaluate the predictive performance and accuracy of this model. The prediction model achieved an area under the ROC curve (AUC) of 0.973 in the training set, with a sensitivity of 93.1% and a specificity of 93.6% (Fig. 3b), while the AUC was 0.949 for the validation set, with a sensitivity of 90.5% and a specificity of 88.6% (Fig. 3c), suggesting favorable discriminatory capacity. Meanwhile, the calibration curves revealed that the model-predicted probability was well consistent with the observed probability, since the bias-corrected curves were very close to the 45-degree ideal lines in both training set (Hosmer-Lemeshow test *P* = 0.832) and validation set (Hosmer-Lemeshow test *P* = 0.659) (Fig. 3d and e). Moreover, to further assess the clinical value of the prediction model, DCA was employed here. The ordinate represented the net benefit, while the abscissa indicated the threshold probability. The results of DCA indicated that the net benefit derived from this model surpassed the “All” and “None” schemes when the threshold probability ranged from 0 to 0.93 in the training set (Fig. 3f) and from 0 to 0.95 in the validation set (Fig. 3g), respectively.

## Discussion

In the present study, we analyzed changes in clinical characteristics and severity of children hospitalized with H1N1 infection in the post-COVID-19 era compared to the pre-COVID-19 era, and constructed a simple and practical risk model integrating eight common demographic, clinical and laboratory indicators for predicting severe H1N1 infection in the post-COVID-19 era.

After PSM for age, gender, and BMI between the pre- and post-COVID-19 groups, we found significant differences in clinical and laboratory indicators among the two groups, including longer fever duration, higher fever peak, more frequent cough and seizures, elevated levels of inflammatory markers and CK-MB, higher rates of severe cases and mechanical ventilation, as well as longer LOS in the post-COVID-19 group than those in the pre-COVID-19 group, suggesting a greater severity in the post-COVID-19 group. The difference of clinical severity of H1N1 infection between these two periods may be explained by the following three main reasons. The first one is the co-infection with SRAS-CoV-2. Although the critical stage of COVID-19 pandemic has ended, the transmission of SARS-CoV-2 is still ongoing at a low level with ‘mini-wave’ pattern [18]. Echoing this viewpoint, a small number of H1N1 patients co-infected SARS-CoV-2 were observed in post-COVID-19 group in our study. There has been evidence suggesting that co-infections will aggravate viral pathology and clinical severity of patients compared to the single infection of SARS-CoV-2 or influenza A virus by prolonging the virus infection period and impairing neutralizing antibody response [19]. The second one is immunity debt. The lack of exposure to pathogens due to strict NPIs during COVID-19 pandemic resulted in a reduced immune stimulation, thereby weakening the adaptive immunity to specific pathogens [20]. The longer the duration of “pathogens low-exposure” is, the greater the risk of future more severe pandemics due to an increasing susceptible population and decreased level of herd immunity [6]. This issue is particularly concerning in China given that China is one of the countries with the most restrictive and longest-duration NPIs. Thirdly, another non-negligible factor may be the medical history of acute SARS-CoV-2 infection due its ultra-high infection rate (more than 82%) between December 2022 and February 2023 in China [10]. According to Carfì et al. [12], 87.4% of patients who recovered from COVID-19 still exhibited at least one persistent COVID-19-associated symptom within approximately two months after discharge, which was considered to be attributable to direct viral injury or its related inflammatory/immune response. For example, decreased frequency of degranulating virus-specific CD3^+^8^+^ T cells have been observed in some patients who had overcome acute SARS-CoV-2 infection for more than eight months, which may represent declined function of these cells or dysfunction of the immune response [21]. In line with this, in our study, the post-COVID-19 group showed a slight decline in proportion of CD3^+^8^+^ T than the pre-COVID-19 group, although the difference was not significant. Besides, among these persistent COVID-19 symptoms observed in recovered patients, respiratory abnormalities were the most common [12, 22]. This might explain why the cough was more prominent in the post-COVID-19 group than that in the pre-COVID-19 group in our study. An intriguing observation is that, although there were no significant differences in the total number of patients vaccinated against COVID-19, the number of patients who received one dose and those who received two doses among the severe and general H1N1 subgroups of the post-COVID-19 group, the severe subgroup showed a prominently lower proportion of patients with three-dose COVID-19 vaccination than the general subgroup, suggesting that COVID-19 vaccination may offer some clinical protective efficacy against severe H1N1 infection in the post-COVID-19 era. Meanwhile, this further confirms from another aspect that SARS-CoV-2 infection indeed generated direct and indirect effects on the H1N1 infection in the post-COVID-19 era.

Moreover, we found higher incidence of seizures in the post-COVID-19 group compared to the pre-COVID-19 group. The increased incidence of seizures in the post-COVID-19 group may serve as the important evidence of the association between elevated clinical severity of H1N1 infection during the post-COVID-19 era and COVID-19 pandemic dominated by the Omicron variant. Our previous study reported that the Omicron variant showed a relatively high neurotropism, with a higher incidence of seizures in children with Omicron infection than those infected with other SARS-CoV-2 strains, reaching 25.5% [23]. Given the Omicron infection rate of over 82% in China between December 2022 and February 2023 [10], neurological damage that has not fully recovered and/or reactivation of residual virus in bodies of patients who had overcome acute Omicron infection might be the potential factors contributing to the increased incidence of seizures in children with H1N1 infection in the post-COVID-19 era [24, 25]. Meanwhile, it is essential to emphasize that even before the COVID-19 pandemic, H1N1 infection itself could also lead to severe neurological complications, potentially resulting in permanent sequelae [26]. In addition, higher level of CK-MB, which is a well-known marker of myocardial damage, was detected in the patients from the post-COVID-19 group compared to those in the pre-COVID-19 group. This is consistent with Zuin et al. [27], who noted an increased risk of developing myocardial damage in individuals within one year after recovering from SARS-CoV-2 infection. Therefore, in the post-COVID-19 era, special attention should be given to neurological complications (such as seizures) and potential risk of myocardial damage in the clinical management of H1N1-infected children.

Furthermore, this study identified that age, BMI, fever duration, leucocyte count, lymphocyte proportion, proportion of CD3^+^ T cells, TNF-α, and IL-10 were independently associated with occurrence of severe cases in children hospitalized with H1N1 infection during the post-COVID-19 period. First, consistent with the prior knowledge [28, 29], both younger and overweight children are more prone to develop severe form of influenza infection. Second, as we know, during viral invasion, the brain orchestrates evolutionary conserved physiological symptoms, aiming to clear the pathogens and promote the survival of host [30]. Of which, fever is a cardinal symptom of viral infection and triggered by pathogen associated molecules that stimulate the secretion of inflammatory factors by immune cells [31]. This mechanism perhaps partially accounts for why TNF-α and IL-10 could play important roles in predicting occurrence of severe H1N1 infection in this study. There is already evidence suggesting that long fever duration and excessive release of inflammatory factors are closely linked with aggravated sickness behaviors and worse outcomes in patients with severe viral infection [32, 33]. Third, differences regarding leucocyte and its subsets among patients with viral infection also reflect the variance in the severity or progression of disease. Consistent with our find, previous studies have reported an increased leucocyte count and reduced lymphocyte count in patients with severe viral infection compared to non-severe cases, where the underlying mechanism primarily involves imbalance of immune system, including excessive activation of immune cells, hyperinflammation, and direct effects of the virus on lymphocyte recruitment [34–36]. The most crucial change in the decreased lymphocytes induced by viral infection is the decline in CD3^+^ T cells, which are regarded as key players in adaptive immunoreaction against influenza infection due to the potent cytotoxic function in activated CD3^+^8^+^ T cells, the function of producing a series of cytokines and facilitating synthesis and secretion of antibodies in CD3^+^4^+^ T cells, etc. [37]. Noteworthy, as we have mentioned earlier, degranulating virus-specific CD3^+^8^+^ T cells were demonstrated to be reduced in some patients who had overcome acute SARS-CoV-2 infection for over eight months [21]. Therefore, the proportion of CD3^+^ T cells will play a significant role for predicting severe H1N1 infecting during the post-COVID-19 era.

Via integrating these above eight variables, we constructed a simple and practical prediction model, which showed good accuracy and discrimination. Meanwhile, net benefit of using this model was determined by DCA method, which suggested the good clinical practicability. This prediction model may be a very suitable tool for early clinical identification of severe cases among children hospitalized with H1N1 infection during the post-COVID-19 era, aiding clinicians in decision-making. Another important and attracted point is that these eight predictive variables used in our model are very common in clinic and are readily available even in a primary hospital without additional financial cost to patients. During the post-COVID-19 era, this model could hold significant value for the clinical management of pediatric H1N1 patients, especially given the greater severity and increased number of hospitalizations of such patients compared to classical influenza individuals in the pre-COVID-19 era.

This study is subject to several limitations. Due to the retrospective nature of the study, the existence of selection bias and residual confounding variables cannot be excluded despite the application of PSM. Despite the multicenter design, there is a relative limitation in the source and distribution of participants due to the study being conducted solely in Yunnan Province, which may cause an excessively high AUC value of the prediction model here. Besides, this prediction model should be validated by external cohorts, which are absent in our study. Future studies should include larger, multicenter cohorts in a prospective multicenter design.

## Conclusions

Pediatric H1N1 infection during the post-COVID-19 era showed a higher overall disease severity compared to classical H1N1 infection in the pre-COVID-19 period. Meanwhile, cough and seizures were more prominent in children with H1N1 infection during the post-pandemic era. Clinicians should be aware of these changes in H1N1 infection in the post-pandemic era and more attention to such patients is needed in clinical work. Besides, a prediction model based on age, BMI, fever duration, leucocyte count, lymphocyte proportion, proportion of CD3^+^ T cells, TNF-α, and IL-10 was constructed and internally validated here, which showed a good performance for predicting severe H1N1 infection in the post-COVID-19 era.

## Data Availability

No datasets were generated or analysed during the current study.

## Abbreviations

ALT: Alanine transaminase
AST: Aspartate transaminase
BMI: Body mass index
CK-MB: Creatine kinase-MB
CRP: C-reactive protein
DCA: Decision curve analysis
hs-TnT: High-sensitivity troponin T
IL-6: Interleukin 6
IL-10: Interleukin 10
IQR: Interquartile range
LASSO: Least Absolute Shrinkage and Selection Operator
LOS: Length of hospital stay
NPIs: Non-pharmaceutical interventions
PSM: Propensity-score matching
ROC: Receiver operating characteristic
TNF-α: Tumor necrosis factor α
